# The Role of Interleukin-17, Interleukin-23, and Transforming Growth Factor-*β* in Pregnancy Complicated by Placental Insufficiency

**DOI:** 10.1155/2017/6904325

**Published:** 2017-06-15

**Authors:** Dorota Darmochwal-Kolarz, Magdalena Michalak, Bogdan Kolarz, Monika Przegalinska-Kalamucka, Agnieszka Bojarska-Junak, Dariusz Sliwa, Jan Oleszczuk

**Affiliations:** ^1^Department of Gynecology and Obstetrics, Institute of Clinical and Experimental Medicine, Medical Faculty, University of Rzeszow, Rzeszow, Poland; ^2^Department of Obstetrics and Perinatology, Medical University of Lublin, Lublin, Poland; ^3^Medical Faculty, University of Rzeszow, Rzeszow, Poland; ^4^Department of Clinical Immunology, Medical University of Lublin, Lublin, Poland

## Abstract

**Aim:**

The aim of the study was to evaluate the role of Interleukin-17 (IL-17), Interleukin-23 (IL-23), and transforming growth factor-*β* (TGF-*β*) in pregnancy complicated by placental insufficiency and in normal pregnancy.

**Material and Methods:**

The study comprised 34 patients with pregnancy complicated by fetal growth restriction (FGR) associated with preeclampsia (PE), as well as 35 healthy pregnant women. The concentrations of IL-17, IL-23, and TGF-*β* in sera from maternal peripheral blood were determined by an immunoenzymatic assay.

**Results:**

There were higher concentrations of IL-17 in the study group when compared to the controls. In the group of patients with placental insufficiency, the levels of IL-17 positively correlated with systolic blood pressure (*R* = 0.42, *p* < 0.01). The study obtained comparable concentrations of IL-23 in both studied groups. The concentrations of TGF-*β* were significantly lower in pregnancy complicated by the insufficiency of placenta when compared to the controls.

**Conclusions:**

It seems possible that the increased concentrations of IL-17 and the deficiency of TGF-*β* in pregnancy complicated by FGR and PE can be responsible for the activation of inflammatory response observed in PE cases.

## 1. Introduction

Hypertension occurs in 5–10% of all pregnancies and together with postpartum hemorrhage and infections creates a deadly triad of pregnancy complications, responsible for the majority of maternal deaths [1]. The presence of hypertension in pregnancy is dangerous to the fetus, leading to fetal growth restriction (FGR), premature abruption of the placenta, and hypoxia, often causing stillbirth. We should keep in mind that the only effective treatment of preeclampsia is the termination of pregnancy, which makes this complication one of the main causes of iatrogenic prematurity [2]. Every year, due to preeclampsia or eclampsia, over 40,000 women and as many as 500,000 children die. This means that 110 women and over 1600 children die each day [3]. Currently, however, there are more and more indications that preeclampsia is a disease of immune etiology and that immune factors are responsible for both impaired trophoblast implantation and the cascade of events leading to placental insufficiency and FGR in the course of preeclampsia [4–6].

In recent years, in order to clarify the immunological mechanisms responsible for the proper implantation process, the Th1/Th2 paradigm has been extended to the Th1/Th2/Th17 and regulatory T cells (Treg) paradigm [7]. Th17 cells have been recently discovered as a subpopulation of T cells, whose cytokine profile is different from Th1 one and Th2 cells [8]. The main task of Th17 helpers is the production of Interleukin-17. Many studies found an increased proportion of Th17 subpopulations in pregnancies complicated by miscarriage, preterm birth, and preeclampsia [9–11]. Interleukin-17 (IL-17, also known as IL-17A) is a major, strongly proinflammatory cytokine produced by Th17 helper cells [12]. Interleukin-17, a cytokine with potent proinflammatory properties, has a proven role in the development of inflammatory processes, acute immunological graft rejection, and autoimmune diseases. It has also been shown that IL-17 affects the maturation of dendritic cells and inhibits the response from the regulatory T cells (Treg), responsible for the phenomenon of immune tolerance [12].

Interleukin-23, which is produced, among others, by macrophages and dendritic cells, is an important component of the inflammatory response. Together with TGF-*β*1 it stimulates the differentiation of CD4^+^ T cells into Th17 cells [13]. In addition, IL-23 increases the local concentration of matrix metallopeptidase 9 (MMP-9) and stimulates angiogenesis, which makes it an extremely important element in a proper implantation. However, clinical studies showed increased expressions of IL-23 in patients with recurrent pregnancy loss [14]. Its role has been shown in the spread of malignant tumors, as well as in autoimmune diseases. Studies in mice found that animals which are devoid of genes responsible for the production of a subunit of the receptor for IL-23 are definitely more prone to multiple sclerosis and inflammatory bowel diseases [15].

Transforming growth factor-*β* (TGF-*β*) released, among others, by macrophages, neutrophils, platelets, and lymphocytes acts primarily to inhibit the proliferation of B and T lymphocytes and NK cells, reduces the release of proinflammatory cytokines, and inhibits the expression of major histocompatibility complex MHC class II on the antigen-presenting cells [16]. In addition, TGF-*β* is involved in the processes of angiogenesis, wound healing, and repair processes, as well as regulation of the entry of cells onto the apoptotic pathway [17]. The best known protein from the TGF-*β* protein family is TGF-*β*1, which is produced by dendritic cells, white blood cells, and NK cells. It was found that TGF-*β*1 has an immunosuppressive effect on T and B lymphocytes, and the lack of this cytokine may predispose patients to more frequent development of autoimmune diseases, such as systemic lupus or scleroderma [18, 19].

The purpose of our study was to estimate the role of IL-17, IL-23, and TGF-*β* in pregnancy complicated by fetal growth restriction associated with preeclampsia as well as in normal pregnancy.

## 2. Material and Methods

Our study comprised 34 patients with pregnancy complicated by fetal growth restriction associated with preeclampsia admitted to the Department of Obstetrics and Perinatology of the Medical University in Lublin. The diagnosis of preeclampsia was made according to the criteria of* American College of Obstetricians and Gynecologists*. The diagnosis of fetal growth restriction was made when less than 10th percentile fetal weight for gestational age was found during ultrasound examination of patients with preeclampsia. Peripheral blood samples from the study group were taken from pregnant patients before starting a therapy. The control group comprised 35 healthy pregnant women with uncomplicated pregnancy. The study was approved by the Ethics Committee of the Medical University of Lublin.

The immunoenzymatic assays were used to determine sera concentrations of IL-17, IL-23, and TGF-*β*. The assays used kits were produced by the Diaclone Company (Besancon, France). The statistical differences between groups were estimated using Mann-Whitney *U* test, chi-squared test, and Fisher's exact test. Differences were defined as statistically significant at the level of *p* < 0.05. For the correlation analysis Spearman's rank correlation test was performed. Two-tailed *p* values less than 0.05 were considered as statistically significant. STATISTICA 7.1 software (StatSoft Poland, Krakow, Poland) was applied to statistical analysis.

## 3. Results

The concentrations of IL-17 in sera of patients with pregnancies complicated by FGR and preeclampsia were significantly higher when compared to healthy pregnant normotensive women (IL-17: median, 3.9 pg/ml; interquartile ranges, 2.55–5.06 pg/ml, versus median, 2.4 pg/ml; interquartile ranges, 1.78–3.11 pg/ml; *p* < 0.01).

In the group of patients with FGR and preeclampsia, the levels of IL-17 positively correlated with systolic blood pressure (*R* = 0.42, *p* < 0.01).

The concentrations of IL-17 in the control group have increased with the progress of pregnancy (*R* = −0.45, *p* < 0.05). This relationship suggests that in normal pregnancy the concentration of IL-17 gradually increases.

The concentrations of IL-23 in sera of patients with pregnancies complicated by FGR and preeclampsia were significantly higher when compared to healthy pregnant normotensive women (IL-23: median, 1.93 pg/ml; interquartile ranges, 1.37–2.68 pg/ml, versus median, 1.95 pg/ml; interquartile ranges, 1.11–2.84 pg/ml; NS).

Among patients with uncomplicated pregnancies, a negative correlation was found between serum IL-23 and the week of pregnancy, when the blood was collected for the testing (*R* = −0.45, *p* < 0.05). This means that in normal pregnancy the levels of IL-23 gradually decrease with the duration of pregnancy.

**Figure 1:**
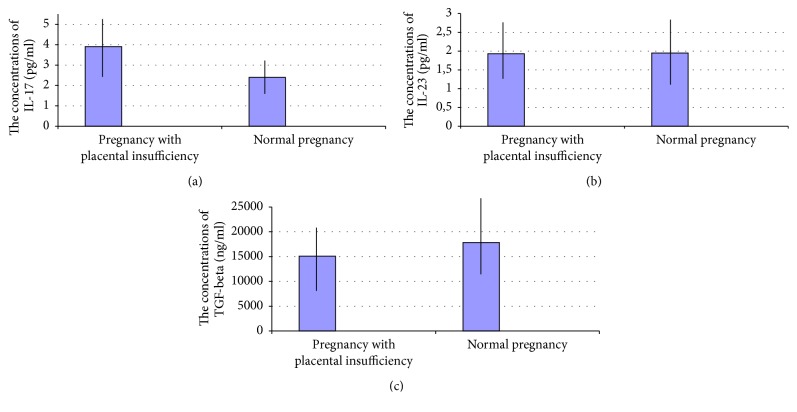
The comparison of (a) IL-17, (b) IL-23, and (c) TGF-*β* concentrations in sera of patients with pregnancy complicated by placental insufficiency (*n* = 34) and healthy women with uncomplicated pregnancy (*n* = 35).

The concentrations of TGF-*β*1 in sera of patients with pregnancy complicated by FGR and preeclampsia were significantly lower when compared to the group of healthy women with uncomplicated pregnancy (TGF-*β*1: median, 15,092 ng/ml; interquartile ranges, 6,801–20,335 ng/ml, versus median, 17,834 ng/ml; interquartile ranges, 12,245–25,395 ng/ml (*p* < 0.05)). The results are presented in Figure 1.

The clinical characteristics of patients from the study and control groups are presented in Table 1.

## 4. Discussion

The mechanisms aimed at maintaining the balance of Th1/Th2/Th17 and Treg cells conditioning the normal development of the pregnancy are not fully understood.

According to Steinborn et al. and Sasaki et al. in pregnancies complicated by placental insufficiency there is a deficit of Treg cells, which supports the expressions of Th17 lymphocytes and the induction of inflammatory response in the fetomaternal interface [20, 21].

The increase in the levels of IL-17 in pregnancy complicated by FGR and PE which was observed in our study confirms the results of our previous study as well as the research conducted by Santner-Nanan et al., who reported a reduction in the number of Treg cells and an increase in the population of Th17 cells in placental complications of pregnancy [9, 22]. On the other hand, during normal pregnancy, the expansions of Treg cells with decreased expressions of Th17 cells have been observed [9, 23].

Furthermore, the results of our study showed that in normal pregnancy the concentrations of IL-17 gradually increases. Interestingly, Martínez-García et al. also noted an increase in the level of IL-17 in the third trimester of uncomplicated pregnancy. The authors attributed an increase in proinflammatory cytokine release near the term of delivery to the dilation of the cervix and the progress of labor [23].

Moreover, we observed that, in the group of patients with FGR and preeclampsia, the concentrations of IL-17 positively correlated with systolic blood pressure. Similar observations were made by Dhillion et al. The authors observed that the administration of IL-17 to healthy pregnant rats resulted in a statistically significant increase in mean arterial blood pressure. The administration of IL-17 to nonpregnant rats had no effect on blood pressure. Interestingly, the increase of blood pressure in pregnant rats was reversible after the administration of superoxide dismutase or the inhibition of angiotensin II receptor type 1, which can mean that the pressure is generated as a result of oxidative stress and the formation of autoantibodies against angiotensin II receptor type 1 [24].

In our study there were no statistically significant differences in the concentrations of IL-23 in the study and control groups. It was noted, however, that there was a negative correlation between the concentrations of IL-23 and the week of pregnancy in which the blood test was collected, suggesting that the concentrations of IL-23 decrease with the duration of pregnancy. The reduced expressions of IL-23 in late physiological pregnancy may be due to the fact that IL-23 plays a central role in early pregnancy, when dendritic cells present antigens of paternal origin conditioning a proper implantation and invasion of trophoblast. A decrease in the concentrations of IL-23 could also explain the tendency of recurrence of certain autoimmune diseases in pregnancy, such as systemic lupus or inflammatory bowel disease [25–28].

Recent studies suggest that IL-23 is the key proinflammatory cytokine secreted by dendritic cells. Antigen-presenting dendritic cells stimulate or inhibit the proliferation of the relevant T cell subpopulations, deciding on the tolerance or rejection of syncytiotrophoblast cells [29]. The action of IL-23 does not result in the differentiation of progenitor cells into the Th1 cells producing interferon-*γ*, but it induces the formation and expansion of Th17, which leads to the release of proinflammatory IL-17 [30]. An interesting observation is the lack of receptors for IL-23 on the surface of undifferentiated Th0 lymphocytes, which are the main effector cells for IL-23. It seems that the receptor expressions of IL-23 occur through the activation of Th0 cells by IL-21 secreted T cells in the presence of IL-6 derived from activated dendritic cells and macrophages. This underlines the importance of dendritic cells presenting the antigens in the induction of inflammatory processes via the expression of IL-23 and, consequently, IL-17 [31, 32].

Moreover, in our study we found decreased expressions of TGF-*β*1 in pregnancy complicated by FGR and PE compared to healthy pregnant women. Contrary to these results, Lygnos et al. observed increased levels of TGF-*β*1 in pregnant women with hypertension [33]. The increased concentrations of TGF-*β*1 were also observed in other pregnancy complications associated with the activation of the inflammatory response, such as miscarriages or premature labor [34]. Transforming growth factor-*β* has immunosuppressive effects and proangiogenic properties. Recently, it has been observed that the conversion of maternal T cells into T CD4^+^FoxP3^+^ regulatory T cells is partially mediated via TGF-*β*. The authors suggest that TGF-*β* can contribute to the fetal-maternal tolerance by the increase of the Treg cell population [35, 36]. It seems possible that the decreased concentrations of TGF-*β*1 observed in pregnancy complicated by FGR and PE can lead to the deficit of Treg cells, Th17/Treg imbalance, and an inappropriate invasion of the trophoblast as well as an abnormal formation of new vessels in the placenta.

## 5. Conclusions

In normal pregnancy the concentrations of IL-17 increase gradually along with the duration of pregnancy, suggesting an increase of the inflammatory activity with the progress of uncomplicated pregnancy. The reduced expressions of IL-23 in late physiological pregnancy may be due to the fact that IL-23 plays a central role in early pregnancy, when dendritic cells present antigens of paternal origin, conditioning proper implantation and the invasion of trophoblast.

Moreover, the increased concentrations of IL-17 and the deficiency of TGF-*β* in pregnancy complicated by FGR and PE can be responsible for the activation of the inflammatory response and as a consequence for a placental insufficiency.

## Figures and Tables

**Table 1:** 

	Placental insufficiency	Normal pregnancy	Statistical significance
Number of cases	34	35	
Age (years)	32.1 ± 4.36	30.7 ± 5.21	NS
First pregnancy	20	14	NS
Another pregnancy	14	21	NS
The duration of gestation (days)	256 ± 33	272 ± 14	0.0002
Time of blood collection (weeks of gestation)	32.05 ± 2.14	32.62 ± 1.63	NS
Vaginal delivery	4	19	0.0018
Caesarean section	30	16	0.0018
Birth weight (g)	2500 ± 1034	3270 ± 453	0.00003
RR systolic (mmHg)	168 ± 14	112 ± 13	0.0001
RR diastolic blood pressure (mmHg)	108 ± 10	72 ± 9	0.0001
Total protein (g/dl)	5.9 ± 0.65	6.3 ± 0.29	0.002
Prothrombin time (s)	10.4 ± 0.5	11.0 ± 0.3	0.00001
Prothrombin index (%)	114.3 ± 7.1	110.2 ± 6.5	0.01
INR	0.87 ± 0.06	0.93 ± 0.06	0.00004
D-dimers (*μ*g/l)	1246 ± 1106	853 ± 600	0.02
Fibrinogen (g/l)	4.8 ± 1.1	5.0 ± 0.9	NS
K (mmol/L)	4.35 ± 0.4	4.0 ± 0.3	0.006
Na (mmol/L)	138 ± 3.4	138 ± 1.6	NS
e-GFR (ml/min/1.73 m^2^)	87.8 ± 37.8	100 ± 32.3	0.02
Creatinine (mg/dl)	0.8 ± 0.3	0.7 ± 0.5	NS
Uric acid (mg/dl)	6.4 ± 1.6	3.8 ± 1.1	0.001
Urea (mg/dl)	23.05 ± 15	16.1 ± 3.8	0.001
